# Incidence, recurrence and management of electrical storm in Brugada syndrome

**DOI:** 10.3389/fcvm.2022.981715

**Published:** 2022-10-25

**Authors:** Ibrahim El-Battrawy, Gretje Roterberg, Jacqueline Kowitz, Assem Aweimer, Siegfried Lang, Andreas Mügge, Xiaobo Zhou, Ibrahim Akin

**Affiliations:** ^1^First Department of Medicine, Faculty of Medicine, University Medical Centre Mannheim, University of Heidelberg, Heidelberg, Germany; ^2^DZHK (German Center for Cardiovascular Research), Mannheim, Germany; ^3^Bergmannsheil Bochum, Medical Clinic II, Department of Cardiology and Angiology, Ruhr University, Bochum, Germany

**Keywords:** Brugada, outcome, electrical storm, complications, cardiac death

## Abstract

**Background:**

Brugada syndrome (BrS) is associated with ventricular tachyarrhythmias. However, the presence of electrical strom (ES) and its management still debated.

**Objectives:**

We present the outcome and management of 44 BrS patients suffering from ES.

**Methods:**

A systematic literature review and pooled analysis Through database review including PubMed, Web of Science, Cochrane Libary and Cinahl studies were analyzed. Evidence from 7 reports of 808 BrS patients was identified.

**Results:**

The mean age of patients suffering from ES was 34 ± 9.5 months (94.7% males, 65.8% spontaneous BrS type I). Using electrophysiological study ventricular tachycardia/ventricular fibrillation were inducible in 12/23 (52.2%). Recurrence of ES was documented in 6.1%. Death from ES was 8.2% after a follow-up of 83.5 ± 53.4. In up to 27 ES resolved without treatment. External shock was required in 35.6%, internal ICD shock in 13.3%, Overdrive pacing, left cardiac sympathetic block and atropin in 2.2%. Short-term antiarrhythmic management was as the following: Isopreterenol or Isopreterenol in combination with quinidine 35.5%, orciprenaline in 2.2%, quinidine 2.2%, disopyramide 2.2% or denopamide 2.2%. However, lidocaine, magensium sulfate, mexiletine and propanolol failed to control ES.

**Conclusion:**

Although ES is rare in BrS, this entity challenges physicians. Despite its high mortality rate, spontaneous termination is possible. Short-term management using Isoproterenol and/or quinidine might be safe. Prospective studies on management of ES are warranted.

## Introduction

Sudden cardiac death (SCD) could be caused by a non-structural heart disease. Brugada syndrome (BrS) belongs non-structural heart disease affecting the sodium channel current. It presents typical ECG findings such as coved ST-segment elevation in at least one precordial lead (≥2 mm). BrS patients are at high risk of malignant tachyarrhythmias (1, 2). In up to 30% of BrS mutations are found with a predominance of voltage-dependent sodium channels (SCN5A). Fever and sodium-channel blockers are known triggers of BrS (3, 4, 12). Therefore, consensus papers have recommended avoiding sodium-channel blocker or fever and by agressive and early treatment of fever (5).

Hydroquinidine (HQ) treatment and catheter ablation therapy might be effective in selected cases in patients with recurrent ventricular arrhythmias (6). Suffering from a prior SCD or syncopal events are predictors for recurrent ventricular tachyarrhythmias and therefore they should be prevented by ICD implantation (7). ICD implantation for primary prevention in BrS patients is controversial. A programmed ventricular stimulation (PVS) may be considered for risk stratification of BrS (7–9). Inducibility of a sustained ventricular tachyarrhythmias among BrS patients presenting type I is a further predictor for ventricular tachyarrhythmias (10). Of note, ICD is not adequate for every patient especially in cases of low risk of developing ventricular tachyarrhythmias over follow-up.

Studies in animals and several cellular models have suggested a voltage gradient in the early phase of repolarization as a part of the ECG phenotype in BrS. A notched phase 1 of the right ventricular outflow tract myocardial action potential may explain a part of the pathomechanism of J-wave in BrS (11). This is related to a loss of function sodium channel current (12). As a part of mode-of mechanism of recurrent ventricular fibrillation might be the previously described premature ventricular beat causing a short-QT-short sequence (13). and may be explained by a mechanism similar to that of the J waves observed in BrS. In addition, it seems that BrS patients, who are suffering from ES show more early repolarization pattern in the ECG.

We aimed to the prognosis and treatment approach of 44 BrS patients suffering from electrical storm (ES).

## Methods

In this analysis, we include all patients diagnosed with BrS and suffering from ES from 2007 to 2018. Only 7 studies showed evidence focusing on ES in BrS (13–18). A total of 7 studies were identified through a systematic database analysis (PubMed, Web of Science, Cochrane Libary, Cinahl) and their data was analyzed according to our model. We used the PICO strategy to identify significant literature by using controlled search items [(Brugada) AND (syndrome)] related to our clinical question (19). Three independent researchers did cross checks on the established database by comparing the collected data. The statistical analysis was performed using SPSS version 25 (IBM, Italy) and the PRISMA-IPD statement checklist was used as guideline to verify the systematic literature review (20).

A coved type 1 BrS pattern in precordial leads either at baseline or after the administration of a sodium channel blocking agent was relevant to confirm the diagnosis. The definition of type 1 ECG has been described and modified before (7, 21). If a non-type 1 BrS was shown administration of a sodium channel blockers was done.

### Data collection of different studies

Several data were extracted from the published papers including age, gender, family history and symptoms. In case of drug testing results of drug testing, results of electrophysiological study and the genetic screening were extracted. The indication of ICD implantation including several predictors for SCD was evaluated.

### Systematic literature review

**Figure 1:**
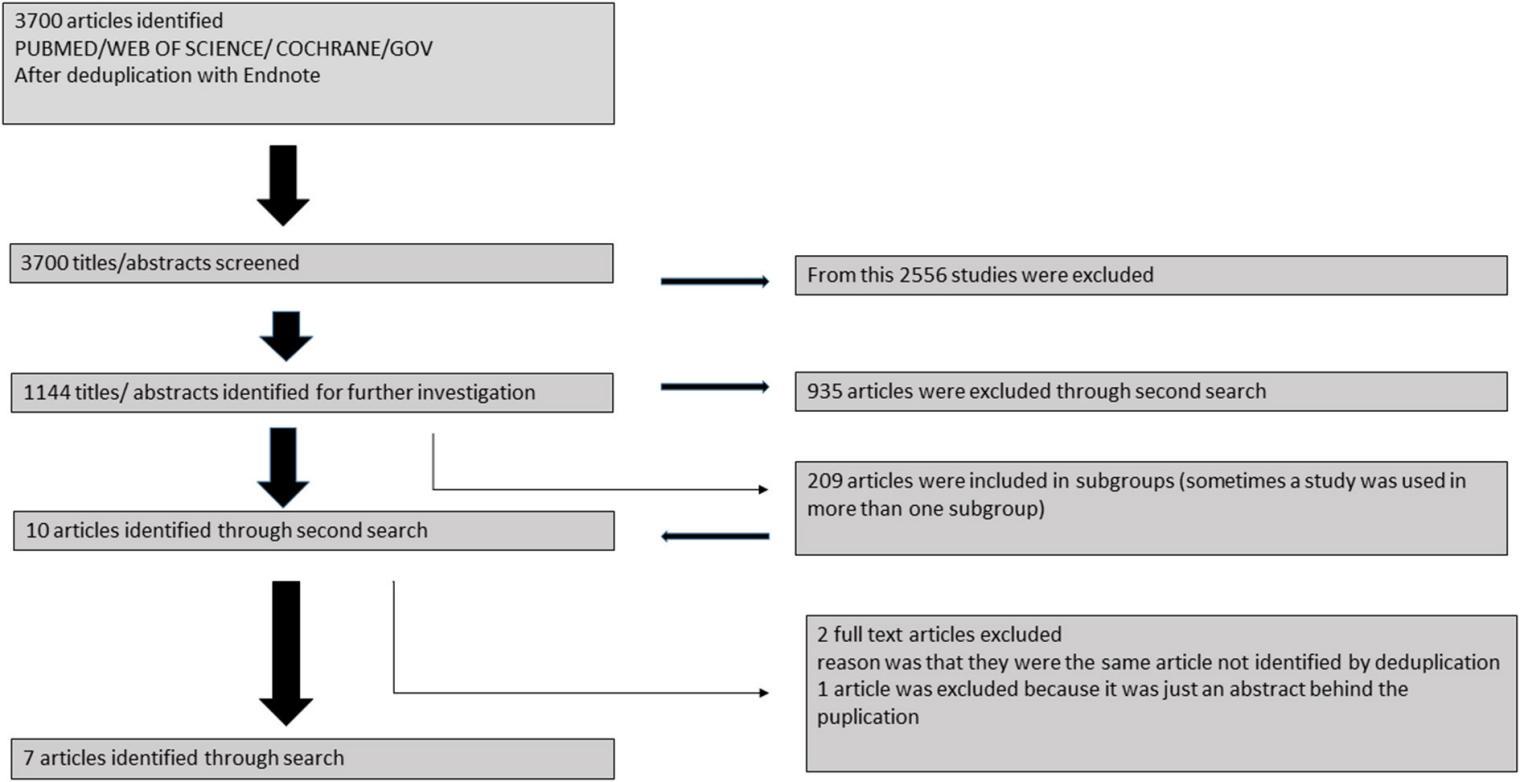
Flowchart of recruitment criteria of the present study. Finally 44 patients were included from 7 studies.

PubMed, Web of Science, Cochrane Libary and Cinahl were screened for BrS and electrical storm (Figure 1). Published reports up to 2018, which were published in English language were taken into consideration. Case reports or studies not reporting on ES were excluded.

### Statistics

Data are presented as mean ± SD for continuous variables with a normal distribution, median (interquartile range) for continuous variables with a non-normal distribution, and as frequency (%) for categorical variables. The Kolmogorov–Smirnov test was used to assess normal distribution. Student's *t*-test and the Mann–Whitney *U*-test were used to compare continuous variables with normal and non-normal distributions, respectively. The Chi-squared-test or Fisher's exact test was used to compare categorical variables.

## Results

### Demographics

The mean age of patients suffering from ES was 34 ± 9.5 years with a predominance of males (94.7%). 65.8% of patients showed spontaneous BrS type I. Symptoms were documented as the following: prior VF was (42.1%), syncope (15.8%).

Inducibility of ventricular tachycardia/ventricular fibrillation was documented in 52.2% of cases. 67.3% of patients had an ICD being implanted before event, Table 1.

**Table 1:** Overview of baseline characteristics of BrS patients receiving ICD implantation and suffering from ES.

**Study**	**Kaneko 2014 *N* = 22**	**Ohgo 2007 *n* = 7**	**Conte 2015 *n* = 4**	**Corcia 2018 *n* = 3**	**Sacher 2013 *n* = 11**	**Schukro 2010 *n* = 1**	**Hernandez–Ojeda 2017 *n* = 1**	**Total *n* = 49**
Number of the cohort	22	76	176	35	378	17	104	808
**Demographics**
Male, *n* (%)	22 (100)	6 (85.7)	3 (75)	3 (100)	n.d.	1 (100)	1 (100)	36 (94.7)
Age	37.5	49.5	41	20.3	n.d.	40	34	34 ± 9.5
BrS type 1, *n* (%)	17 (77.3)	4 (57.1)	2 (50)	1 (33.3)	n.d.	1 (100)	0 (0)	25 (65.8)
**Symptoms**, ***n*** **(%)**
Prior VF or SCD	6 (27.3)	4 (57.1)	4 (100)	1 (33.3)	n.d.	1 (100)	0 (0)	16 (42.1)
Prior syncope	0 (0)	3 (42.9)	0 (0)	2 (66.7)	n.d.	0 (0)	1 (100)	6 (15.8)
Asymptomatic	16 (72.7)	0 (0)	0 (0)	0 (0)	n.d.	0 (0)	0 (0)	16 (42.1)
**Electrophysiological study data**, ***n*** **(%)**
Inducible VF or VT	6/11 (54.5)	4/7 (57.1)	n.d.	1/3 (33.3)	n.d.	1/1 (100)	0/1(0)	12/23 (52.2)
**ICD implantation**, ***n*** **(%)**
Prior ICD implantation	6 (27.3)	7 (100)	4 (100)	3 (100)	11 (100)	1(100)	1(100)	33 (67.3)
ICD implantation after ES	16 (72.7)	0 (0)	0 (0)	0 (0)	0 (0)	0(0)	0 (0)	16 (32.7)
**Follow-up data**
Recurrence of ES, n (%)	0 (0)	1 (14.3)	1 (25)	0 (0)	0 (0)	1 (100)	0 (0)	3 (6.1)
Death because of ES	0 (0)	0 (0)	0 (0)	3 (100)	0 (0)	0 (0)	1 (100)	4 (8.2)
Follow-up time, mean (months)	76.8 ± 60	114 ± 57.6	83.8 ± 57.3	115 ± 56.4	77 ± 42	57 ± 32.3	18	83.5 ± 53.4

ICD, implantable cardioverter defibrillator; n.d., no information; VF, ventricular fibrillation; VT, ventricular tachycardia.

### Short-term management of ES

Whereas, external shock was required in 35.6%, internal ICD shock was required in 13.3%. Interestingly in 26.7% of patients ES resolved without treatment. Furthermore, the use of different drugs was documented with a high presence of Isoproterenol 33.3%, Table 2. Other drugs such as lidocaine, mexiletine, propafol and magnesium sulfate were ineffective.

**Table 2:** Management of BrS suffering from ES.

**Study**	**Kaneko 2014 *n* = 22**	**Ohgo 2007 *n* = 7**	**Conte 2015 *n* = 4**	**Corcia 2018 *n* = 3**	**Sacher 2013 *n* = 11**	**Schukro 2010 *n* = 1**	**Hernandez–Ojeda 2017 *n* = 1**	**Total number *n* = 44**
**Effective short-term management**, ***n*** **(%)**								
External shock	16 (72.7)	0 (0)	n.d.	0 (0)	0 (0)	0 (0)	0 (0)	16 (35.6)
Internal ICD shock	6 (27.3)	0 (0)	n.d.	0 (0)	0 (0)	0 (0)	0 (0)	6 (13.3)
Overdrive pacing. left cardiac sympathetic block and atropine	1 (4.5)	0 (0)	n.d.	0(0)	0 (0)	0 (0)	0 (0)	1 (2.2)
Oral disopyramide	1 (4.5)	0 (0)	n.d.	0 (0)	0 (0)	0 (0)	0 (0)	1 (2.2)
Isoprotenerol	6 (27.3)	5 (71.4)	n.d.	0 (0)	4 (36.4)	0 (0)	0 (0)	15 (33.3)
Isoprotenerol and quinidine	1 (4.5)	0 (0)	n.d.	0 (0)	0 (0)	0 (0)	0 (0)	1 (2.2)
Onciprenalide + ICD	0 (0)	0 (0)	n.d.	0 (0)	0 (0)	1 (100)	0 (0)	1 (2.2)
Resolved sponatous (no treatment)	12 (45.5)	0 (0)	n.d.	0 (0)	0 (0)	0 (0)	0 (0)	12 (26.7)
Catheter ablation	1 (4.5)	0 (0)	n.d.	0 (0)	0 (0)	0 (0)	0 (0)	1 (2.2)
Denopamide	0 (0)	1 (14.3)	n.d.	0 (0)	0 (0)	0 (0)	0 (0)	1 (2.2)
Quinidine	0 (0)	1 (14.3)	n.d.	0 (0)	0 (0)	0 (0)	0 (0)	1 (2.2)
**Ineffective short-term management**, ***n*** **(%)**								
Lidocaine	4 (18.2)	0 (0)	n.d.	0 (0)	0 (0)	0 (0)	0 (0)	4 (11.4)
Magnesium sulfate	3 (13.6)	0 (0)	n.d.	0 (0)	0 (0)	0 (0)	0 (0)	3 (6.7)
Propanolol	2 (9.1)	0 (0)	n.d.	0 (0)	0 (0)	0 (0)	0 (0)	2 (4.4)
Mexilitine	1 (4.5)	0 (0)	n.d.	0 (0)	0 (0)	0 (0)	0 (0)	1 (2.2)
Internal and external defibrillation	0 (0)	0 (0)	n.d.	1 (33.3)	0 (0)	0 (0)	0 (0)	1 (2.2)
Internal defibrillation	0 (0)	0 (0)	n.d.	1 (33.3)	0 (0)	0 (0)	0 (0)	1 (2.2)
Unknown	0 (0)	0 (0)	n.d.	1 (33.3)	0 (0)	0 (0)	1(100)	2 (4.4)
**Long-term management**, ***n*** **(%)**								
Bepridil (100–200 mg)	6 (27.3)	0 (0)	0 (0)	n.p.	0 (0)	0 (0)	n.p.	6 (13.3)
Quinidine	2 (9.1)	0 (0)	2 (50)	n.p.	6 (54.5)	1 (100)	n.p.	11 (24.4)
Amiodarone	1 (4.5)	0 (0)	0 (0)	n.p.	0 (0)	0 (0)	n.p.	1 (2.2)
Disopyramide	1 (4.5)	0 (0)	0 (0)	n.p.	0 (0)	0 (0)	n.p.	1 (2.2)
Denopamine	0 (0)	3 (42.9)	0 (0)	n.p.	0 (0)	0 (0)	n.p.	3 (6.7)
Denopamide+ Quinidine+ Isoproerenol	0 (0)	1 (14.3)	0 (0)	n.p.	0 (0)	0 (0)	n.p.	1 (2.2)
Denopamide + Quinidine	0 (0)	1 (14.3)	0 (0)	n.p.	0 (0)	0 (0)	n.p.	1 (2.2)
Quinidine + Cilostazol+ Bepridil	0 (0)	1 (14.3)	0 (0)	n.p.	0 (0)	0 (0)	n.p.	1 (2.2)
Catheter ablation	0 (0)	0 (0)	1 (25)	n.p.	5 (45.5)	0 (0)	n.p.	6 (13.3)
Heart transplantation	0 (0)	0 (0)	1 (25)	n.p.	0 (0)	0 (0)	n.p.	1 (2.2)
No drug treatment	12 (54.5)	2 (28.6)	0 (0)	n.p.	0 (0)	0 (0)	n.p.	13 (28.9)
Ineffective long-term drug management with VF recurrence, *n* (%)	12 (54.5)	5 (71.4)	0 (0)	n.p.	0 (0)	0 (0)	n.p.	17 (37.8)
Follow-up time. mean (months)	76.8 ± 60	114 ± 57.6	83.8 ± 57.3	115 ± 56.4	77 ± 42	57 ± 32.3	18	83.5 ± 53.4

### Long-term management of ES

Catheter ablation of VT was documented in 13.3%. Following drugs were used bepridil (*n* = 6), quinidine (*n* = 11), amiodarone (*n* = 1), disopyramide (*n* = 1), denopamide (*n* = 1), denopamide + HQ + Isoproerenol (*n* = 1), denopamide + HQ (*n* = 1), quinidine + cilostazol + bepridil (*n* = 1). Interestingly one patient received heart transplantation. Overall recurrence of ES was 6.8% and overall death regarding ES was 9.1%.

## Discussion

We describe the short- and long-term incidence and management of ES in BrS patients from 7 defined studies after a systematic literature review and found the following

(i) ES in BrS is resolving spontaneously in one third of cases.
(ii) Up to 6.1 and 8.2% of patients suffer from recurrence of ES and death; respectively.
(iii) Different drugs might be useful for short-term and long-term treatment of ES with highest presence of Isoproterenol and HQ; however catheter ablation could be another treatment strategy.

Although ES is a rare finding in BrS patients, this issue is important regarding its mortality nature. The mean age of sudden death in BrS is predominantly documented at the young with a mean age of 41 ± 15 years (22). Up to 18–30% of BrS account to mutations in the SCN5A. However, the majority of patients do not show any mutation (22–25).

Recently published data showed 3-fold increased mortality risk of patients suffering from ES (26). Even more, it has been reported about 20% increased mortality rate per shock episode. Additionally multiple shock increase the risk of acute heart failure and consequently mortality rate (27).

The present study in BrS suffering from ES presents a mortality rate of 8.2% over mean follow-up of 83.5 months. This low mortality rate might be related to different factors such as spontaneous the termination of ES without treatment in up to 27% of patients. One important finding of our study is that the majority of patients suffering from ES are presenting spontaneously BrS type 1 ECG. Even more 42.1% of patients have had a prior VF and/or VT. Therefore, these data provide a further support for the requirement of ICD placement in Brugada patients with previous episodes of arrhythmic events.

Another important data point is the inducibility of VT and/or VF in BrS patients. Our data analysis shows that more than 52.2% of patients suffering from ES have inducible sustained ventricular tachyarrhythmias, which means that this cohort is a high risk cohort. Among patients with ES at least 42% of patients suffered from VT/VF in the past.

Sroubek et al. reported in a pooled analysis of 1,312 BrS patients, that in 527 patients arrhythmias were induced with programmed ventricular stimulation (8). Induction was associated with cardiac events during follow-up [hazard ratio, 2.66; 95% confidence interval (CI), 1.44–4.92, *P* < 0.001]. This risk was greatest with lowest extrastimuli (1–2 extrastimuli). Absence of syncope or presence of type 1 BrS ECG during sodium channel blocker use were associated with lowest risk of sudden cardiac death over follow-up. Therefore, when arrhythmias are induced within electrophysiology work-up other clinical factors should be taken into consideration before ICD implantation.

Different treatment strategies have been investigated in patients suffering from ES such as sympathetic blockade, different beta-blockers including propranolol, esmolol, and left stellate ganglionic blockade with combined therapy with Class I antiarrhythmic drugs (lidocaine, procainamide, and bretylium). Additionally, the combination of amiodarone and propranolol showed an improvement of survival rate (28, 29). However, BrS present a rare entity with different treatment strategy regarding triggering the risk of life-threatening arrhythmia with use of class I anti-arrhythmic drugs and beta-blockers. Whereas, reducing the sympathic activity is a sole treatment of patients suffering from ES, in BrS patients the use of sympatic triggers including isoproterenol are successfully for acute management of ES. Quinidine has been reported to reduced rate of ICD shock over follow-up among BrS patients (30).

Of note, the use of catheter ablation in the present cohort with 13% is low. This might be related to absence of arrhythmic events after acute and long-term use of anti-arrhythmic drugs. In addition, ablation in BrS might need a well-expertise, which may explain the low-rate use of this procedure in the past. Furthermore, the epicardial ablation procedure has been firstly well-described the last 7 years, whereas the included papers are in particular older. Of note, the reported guidelines by Priori et al. (7) classified the use of catheter ablation due to ES as class IIb. It is important to emphasize more robust cohorts of BrS undergoing epicardial ablation were reported the recent years. It was reported that these patients have unique abnormal low voltage, prolonged duration, and fractionated late potentials mainly in the anterior aspect of the right flow tract epicardium (31).

On the other hand isoproterenol use is recommend in BrS patients within ES (class IIa recommendation) (7). Regarding the mode of action of isoproterenol among BrS patients during ES published data 1999 suggested (32) that phase 2 re-entry is a part of mechanism of ventricular tachyarrhythmias among BrS patients. Either increased outward current or decreased inward current induces a change in the epicardial action potential, e.g., deepening of phase 1 notch and shortening of action potential duration, and excitation propagates as a difference in electrical voltage (phase 2 re-entry) (33). The stimulation of beta-adrenergic receptors, e.g., by use of isoproterenol causes increased inward calcium current and reduces the excess of outward current, consistent with changes in action potential characteristics.

### Study limitation

This study provides registry data dominated by retrospective studies and, although the authors clinically evaluated all patients, clinical assessment and treatment algorithm was not uniform and consecutively ICD indications were heterogeneous throughout the study, some patients had an ICD implanted for reasons other than symptoms or ventricular fibrillation inducibility. The treatment of ES was not related to similar protocol.

Moreover, despite advantages of recruited ablation studies as a novel therapeutic approaches, this issue was not deeply analyzed in this study.

Finally, we present in this paper a pooled analysis of different study groups, therefore bias are not excluded.

## Conclusions

Although ES is rare in BrS, this entity challenges physicians. Using of sympathicus triggers and the off-lable use of HQ might be successful in short- and long-management of in BrS.

## Data availability statement

The original contributions presented in the study are included in the article/supplementary material, further inquiries can be directed to the corresponding author/s.

## Ethics statement

The requirement of ethical review and approval was waived. The requirement of written informed consent from the patients/participants was waived.

## Author contributions

IE-B: writing the first draft. GR and IE-B: data analysis. SL and XZ: supervision. SL and GR: statistical review. IA, AM, and AA: critical revision. All authors contributed to the article and approved the submitted version.

## Conflict of interest

The authors declare that the research was conducted in the absence of any commercial or financial relationships that could be construed as a potential conflict of interest.

## Publisher's note

All claims expressed in this article are solely those of the authors and do not necessarily represent those of their affiliated organizations, or those of the publisher, the editors and the reviewers. Any product that may be evaluated in this article, or claim that may be made by its manufacturer, is not guaranteed or endorsed by the publisher.
